# Retraction: Signal processing for enhancing railway communication by integrating deep learning and adaptive equalization techniques

**DOI:** 10.1371/journal.pone.0345999

**Published:** 2026-04-08

**Authors:** The PLOS One Editors

The *PLOS One* Editors retract this article [1] due to concerns about potential manipulation of the publication process. These concerns call into question the validity and provenance of the reported results. We regret that the issues were not identified prior to the article’s publication.

All authors either did not respond directly or could not be reached.

## References

[1] Wang Y, Chang W, Li J, Yang C. RETRACTED: Signal processing for enhancing railway communication by integrating deep learning and adaptive equalization techniques. PLoS One. 2024;19(10):e0311897. doi: 10.1371/journal.pone.0311897 39392828 PMC11469544

